# Response to: Comment on “Exotropia Is the Main Pattern of Childhood Strabismus Surgery in the South of China: A Six-Year Clinical Review”

**DOI:** 10.1155/2017/7402715

**Published:** 2017-03-16

**Authors:** Xinping Yu, Zhouduo Ji, Huanyun Yu, Meiping Xu, Jinling Xu

**Affiliations:** ^1^The Eye Hospital of Wenzhou Medical University, Wenzhou 325027, China; ^2^Ophthalmology Department, Zhengzhou Second Hospital, Zhengzhou 450006, China

Thanks are due to Dr. Ayyildiz et al. [1] for the constructive questions and suggestions to our publication [2]. We included our answers below.

The distributions of strabismus subtypes for all age groups between 2006 and 2011 were listed in Table 1.

The surgical criteria for intermittent exotropia were the same for all patients included in the study, which included (1) the degree of deviation (distance and/or near) ≧20 PD and (2) the frequency of deviation occurring more than 1/2 of waking hours, and/or (3) those combined with sensory deficit [3, 4]. Here the sensory deficit included the stereoacuity at near and distance, binocular vision measured through stereoscope, and the sensory fusion measured by Worth 4 Dot test.

And the surgical criteria for esotropia in children are mainly based on the effect of deviation with wearing corrected glasses, in patients still with deviation more than 10 PD at near or distance with the corrected glasses [5].

We did evaluate the stereoacuity at distance using Optec 3500 in the study. 722 (32.5%) subjects had 40–480 seconds of arc at distance, 996 (44.8%) had >480 seconds of arc, and 505 (22.7%) were uncooperative to the test.

We hope we have addressed all questions and made our result clearer.

## Figures and Tables

**Table 1:** Distributions of strabismus subtypes for all age groups between 2006 and 2011.

Age group	Year											
	2006		2007		2008		2009		2010		2011	
	0–6	7–17	0–6	7–17	0–6	7–17	0–6	7–17	0–6	7–17	0–6	7–17
Intermittent exotropia	6	45	5	36	15	67	29	78	31	88	40	115
	(10%)	(24%)	(6%)	(17%)	(16%)	(25%)	(28%)	(29%)	(24%)	(29%)	(25%)	(33%)
Constant exotropia	15	27	8	57	10	51	17	71	13	49	14	61
	(23%)	(15%)	(9%)	(28%)	(11%)	(19%)	(16%)	(26%)	(10%)	(16%)	(9%)	(17%)
Infant esotropia	3	9	14	7	8	6	7	5	10	14	15	13
	(5%)	(5%)	(16%)	(3%)	(8%)	(2%)	(6.5%)	(2%)	(8%)	(5%)	(9%)	(4%)
Partial accommodative esotropia	4	12	10	16	9	18	10	11	9	15	16	20
	(6%)	(6%)	(12%)	(8%)	(9%)	(7.5%)	(10%)	(4%)	(7%)	(5%)	(10%)	(6%)
Nonaccommodative esotropia	9	30	14	35	20	48	18	35	14	49	21	40
	(14%)	(16%)	(16%)	(17%)	(21%)	(18%)	(17%)	(13%)	(11%)	(16%)	(13%)	(12%)
Congenital superior oblique palsy	8	8	10	6	14	16	8	12	14	13	13	20
	(12%)	(4%)	(12%)	(3%)	(15%)	(6.5%)	(7.5%)	(4%)	(11%)	(4%)	(8%)	(6%)
Others	19	55	25	50	19	58	16	60	37	76	42	78
	(30%)	(30%)	(29%)	(24%)	(20%)	(22%)	(15%)	(22%)	(29%)	(25%)	(26%)	(22%)

Total	64	186	86	207	95	264	105	272	128	304	161	347
	(100%)	(100%)	(100%)	(100%)	(100%)	(100%)	(100%)	(100%)	(100%)	(100%)	(100%)	(100%)
